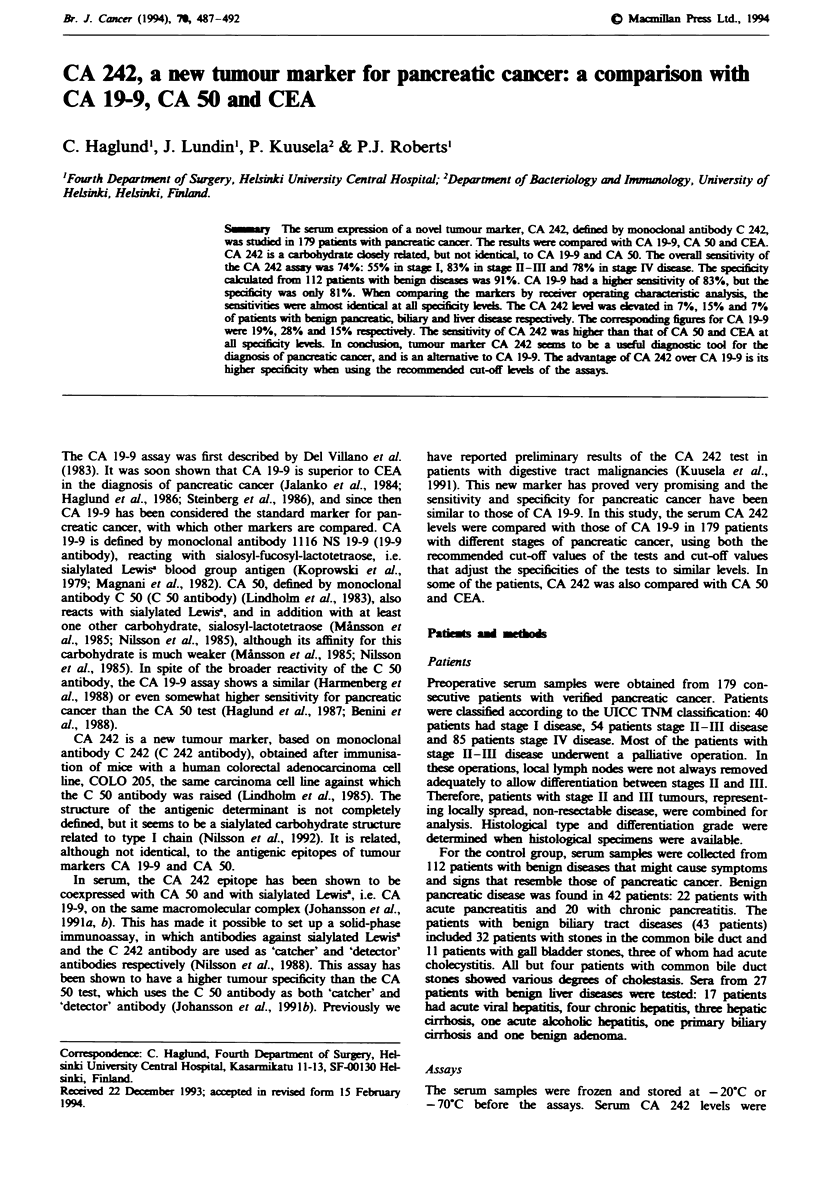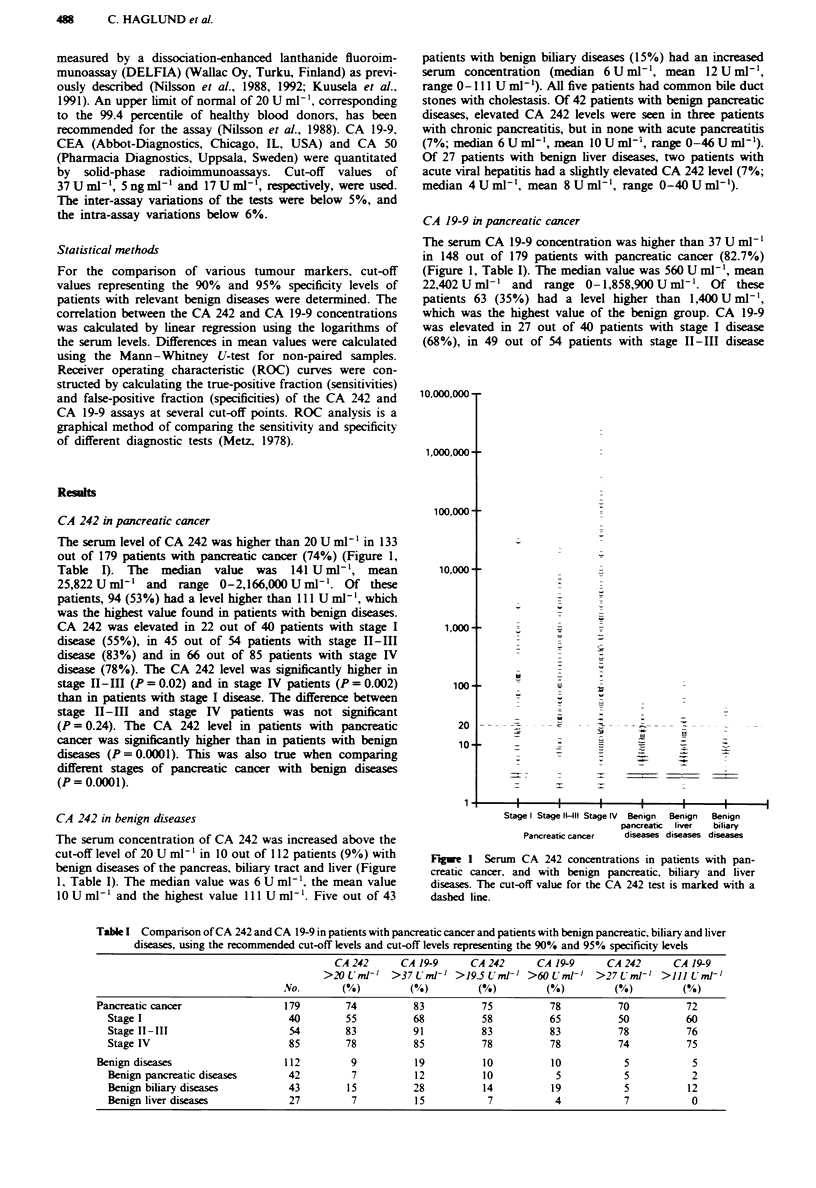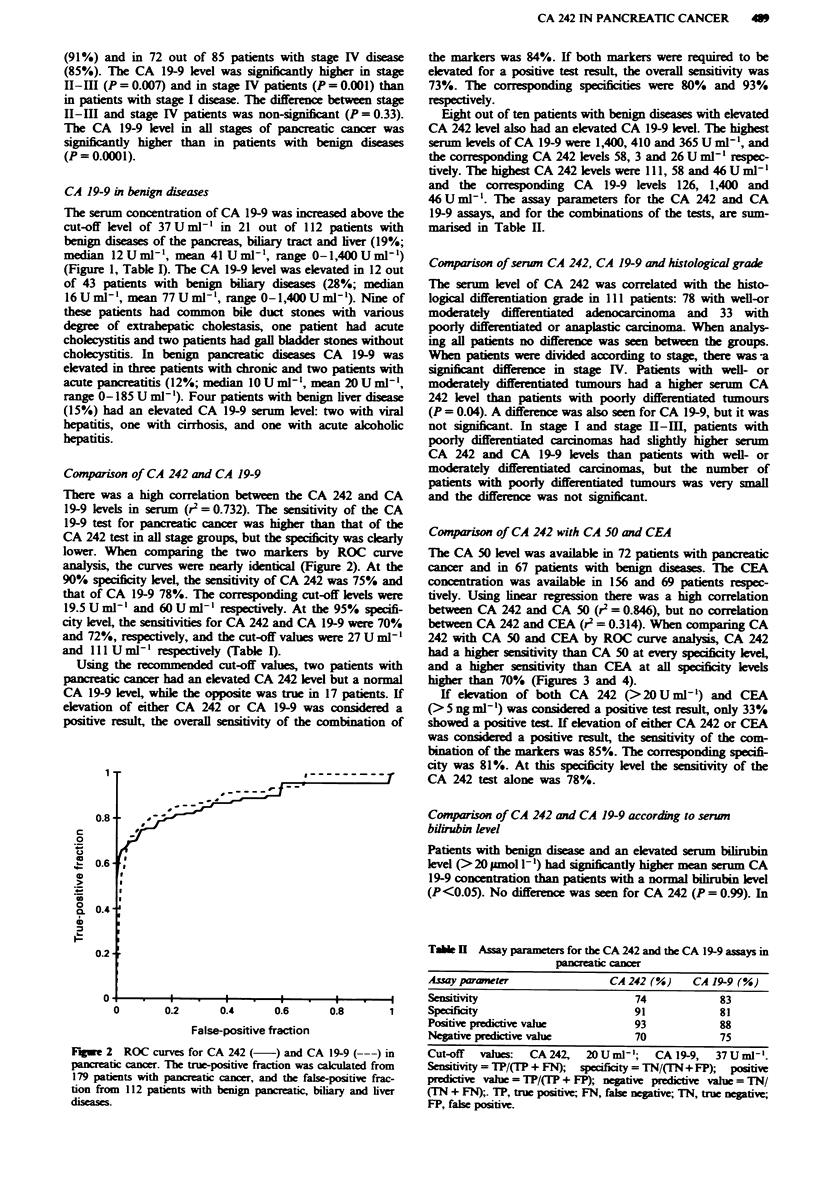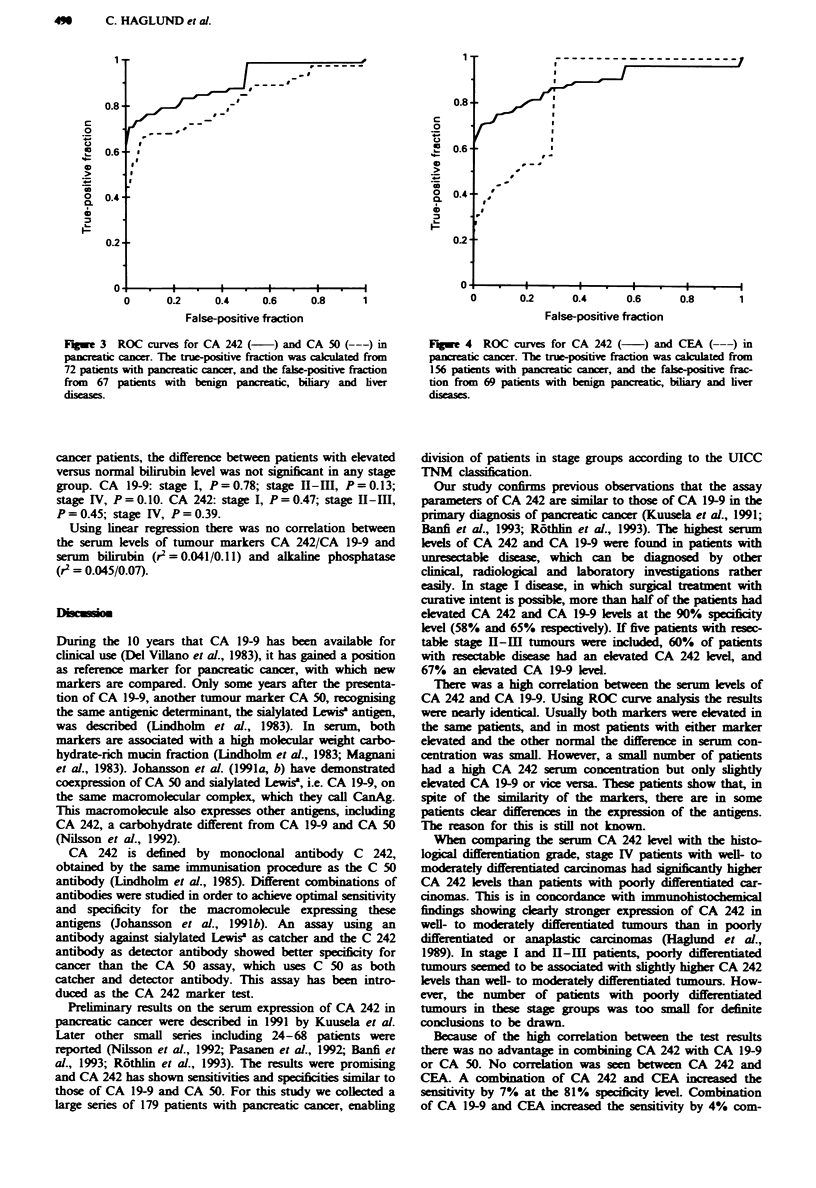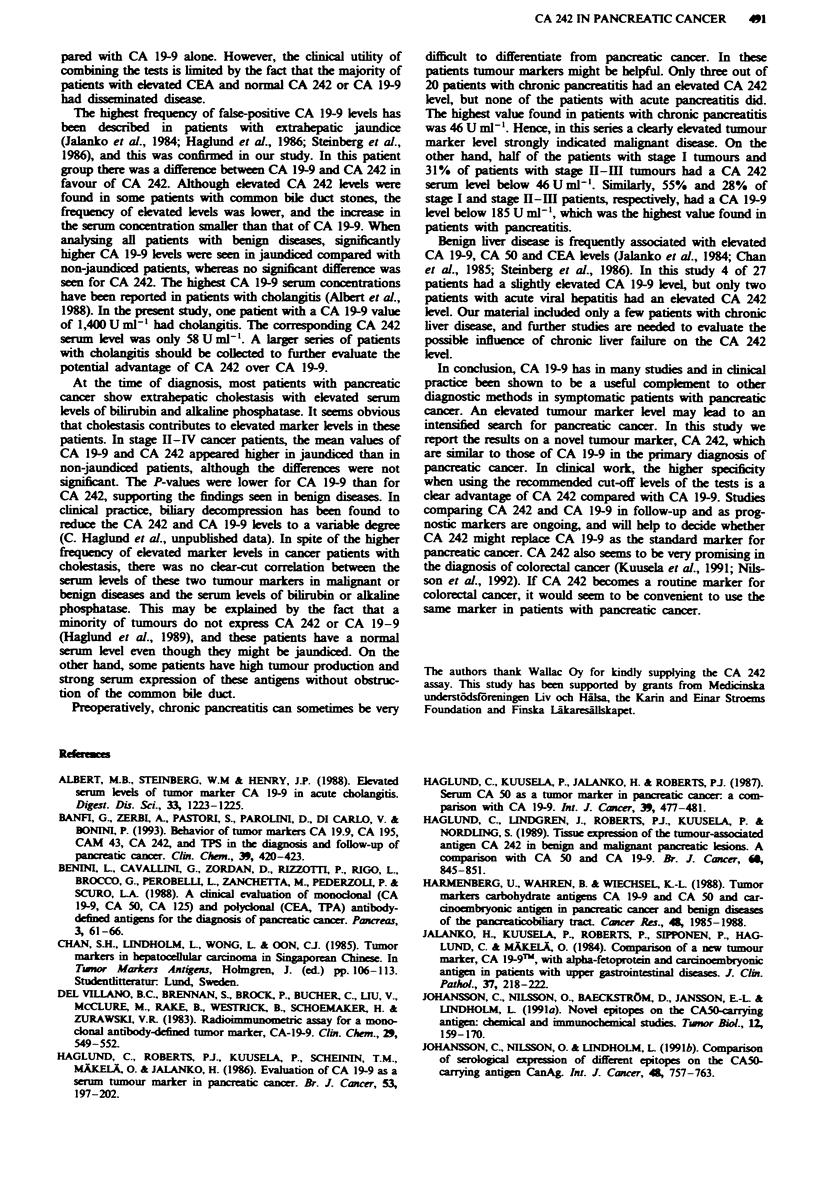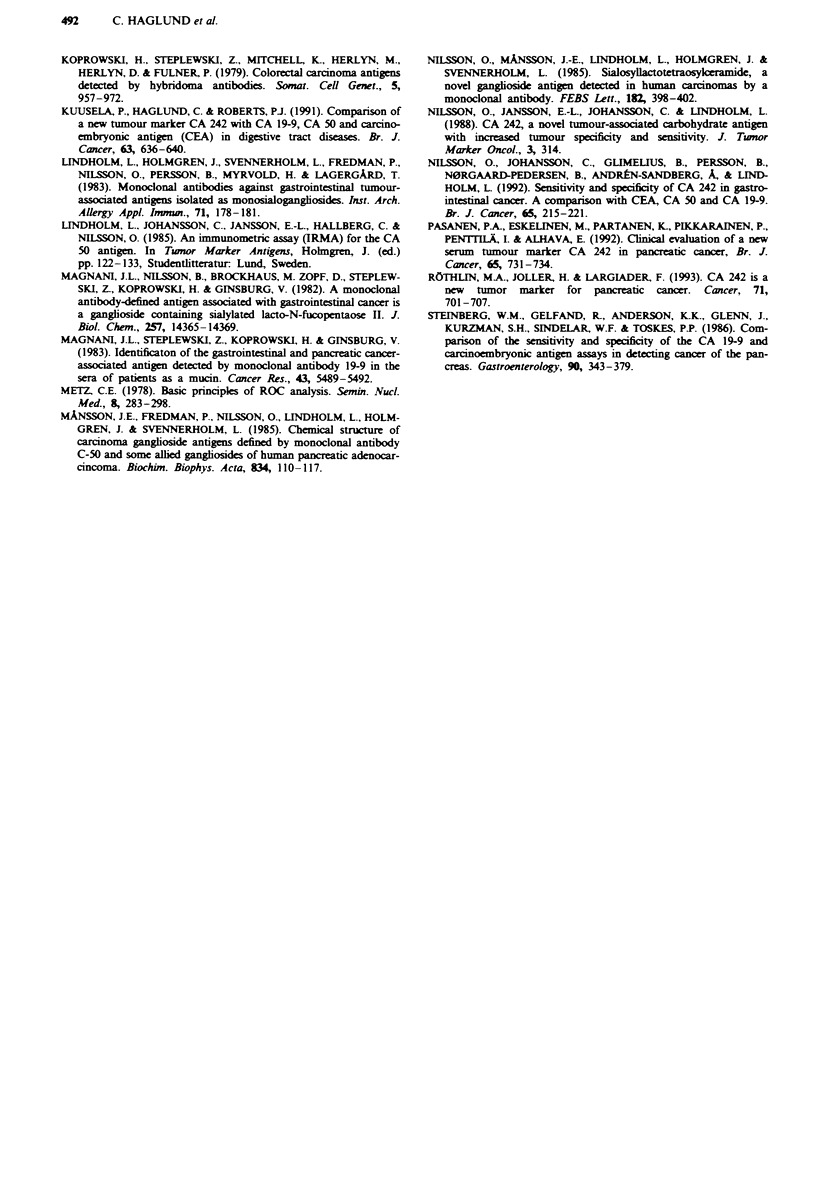# CA 242, a new tumour marker for pancreatic cancer: a comparison with CA 19-9, CA 50 and CEA.

**DOI:** 10.1038/bjc.1994.332

**Published:** 1994-09

**Authors:** C. Haglund, J. Lundin, P. Kuusela, P. J. Roberts

**Affiliations:** Fourth Department of Surgery, Helsinki University Central Hospital, Finland.

## Abstract

The serum expression of a novel tumour marker, CA 242, defined by monoclonal antibody C 242, was studied in 179 patients with pancreatic cancer. The results were compared with CA 19-9, CA 50 and CEA. CA 242 is a carbohydrate closely related, but not identical, to CA 19-9 and CA 50. The overall sensitivity of the CA 242 assay was 74%: 55% in stage I, 83% in stage II-III and 78% in stage IV disease. The specificity calculated from 112 patients with benign diseases was 91%. CA 19-9 had a higher sensitivity of 83%, but the specificity was only 81%. When comparing the markers by receiver operating characteristic analysis, the sensitivities were almost identical at all specificity levels. The CA 242 level was elevated in 7%, 15% and 7% of patients with benign pancreatic, biliary and liver disease respectively. The corresponding figures for CA 19-9 were 19%, 28% and 15% respectively. The sensitivity of CA 242 was higher than that of CA 50 and CEA at all specificity levels. In conclusion, tumour marker CA 242 seems to be a useful diagnostic tool for the diagnosis of pancreatic cancer, and is an alternative to CA 19-9. The advantage of CA 242 over CA 19-9 is its higher specificity when using the recommended cut-off levels of the assays.


Br. J. Cancer (1994), 70, 487-492 © Macmillan Press Ltd., 1994

### CA 242, a new tumour marker for pancreatic cancer: a comparison with CA 19-9, CA 50 and CEA

C. Haglund[1], J. Lundin[1], P. Kuusela[2] & P.J. Roberts[1]

*[1]Fourth Department of Surgery, Helsinki University Central Hospital; [2]Department of Bacteriology and Immunology, University of Helsinki, Helsinki, Finland.*

**Summary** The serum expression of a novel tumour marker, CA 242, defined by monoclonal antibody C 242, was studied in 179 patients with pancreatic cancer. The results were compared with CA 19-9, CA 50 and CEA. CA 242 is a carbohydrate closely related, but not identical, to CA 19-9 and CA 50. The overall sensitivity of the CA 242 assay was 74%: 55% in stage I, 83% in stage II-III and 78% in stage IV disease. The specificity calculated from 112 patients with benign diseases was 91%. CA 19-9 had a higher sensitivity of 83%, but the specificity was only 81%. When comparing the markers by receiver operating characteristic analysis, the sensitivities were almost identical at all specificity levels. The CA 242 level was elevated in 7%, 15% and 7% of patients with benign pancreatic, biliary and liver disease respectively. The corresponding figures for CA 19-9 were 19%, 28% and 15% respectively. The sensitivity of CA 242 was higher than that of CA 50 and CEA at all specificity levels. In conclusion, tumour marker CA 242 seems to be a useful diagnostic tool for the diagnosis of pancreatic cancer, and is an alternative to CA 19-9. The advantage of CA 242 over CA 19-9 is its higher specificity when using the recommended cut-off levels of the assays.

The CA 19-9 assay was first described by Del Villano *et al.* (1983). It was soon shown that CA 19-9 is superior to CEA in the diagnosis of pancreatic cancer (Jalanko *et al.*, 1984; Haglund *et al.*, 1986; Steinberg *et al.*, 1986), and since then CA 19-9 has been considered the standard marker for pancreatic cancer, with which other markers are compared. CA 19-9 is defined by monoclonal antibody 1116 NS 19-9 (19-9 antibody), reacting with sialosyl-fucosyl-lactotetraose, i.e. sialylated Lewis$^a$ blood group antigen (Koprowski *et al.*, 1979; Magnani *et al.*, 1982). CA 50, defined by monoclonal antibody C 50 (C 50 antibody) (Lindholm *et al.*, 1983), also reacts with sialylated Lewis$^a$, and in addition with at least one other carbohydrate, sialosyl-lactotetraose (Månsson *et al.*, 1985; Nilsson *et al.*, 1985), although its affinity for this carbohydrate is much weaker (Månsson *et al.*, 1985; Nilsson *et al.*, 1985). In spite of the broader reactivity of the C 50 antibody, the CA 19-9 assay shows a similar (Harmenberg *et al.*, 1988) or even somewhat higher sensitivity for pancreatic cancer than the CA 50 test (Haglund *et al.*, 1987; Benini *et al.*, 1988).

CA 242 is a new tumour marker, based on monoclonal antibody C 242 (C 242 antibody), obtained after immunisation of mice with a human colorectal adenocarcinoma cell line, COLO 205, the same carcinoma cell line against which the C 50 antibody was raised (Lindholm *et al.*, 1985). The structure of the antigenic determinant is not completely defined, but it seems to be a sialylated carbohydrate structure related to type I chain (Nilsson *et al.*, 1992). It is related, although not identical, to the antigenic epitopes of tumour markers CA 19-9 and CA 50.

In serum, the CA 242 epitope has been shown to be coexpressed with CA 50 and with sialylated Lewis$^a$, i.e. CA 19-9, on the same macromolecular complex (Johansson *et al.*, 1991*a*, *b*). This has made it possible to set up a solid-phase immunoassay, in which antibodies against sialylated Lewis$^a$ and the C 242 antibody are used as 'catcher' and 'detector' antibodies respectively (Nilsson *et al.*, 1988). This assay has been shown to have a higher tumour specificity than the CA 50 test, which uses the C 50 antibody as both 'catcher' and 'detector' antibody (Johansson *et al.*, 1991*b*). Previously we have reported preliminary results of the CA 242 test in patients with digestive tract malignancies (Kuusela *et al.*, 1991). This new marker has proved very promising and the sensitivity and specificity for pancreatic cancer have been similar to those of CA 19-9. In this study, the serum CA 242 levels were compared with those of CA 19-9 in 179 patients with different stages of pancreatic cancer, using both the recommended cut-off values of the tests and cut-off values that adjust the specificities of the tests to similar levels. In some of the patients, CA 242 was also compared with CA 50 and CEA.

Correspondence: C. Haglund, Fourth Department of Surgery, Helsinki University Central Hospital, Kasarmikatu 11-13, SF-00130 Helsinki, Finland.

Received 22 December 1993; accepted in revised form 15 February 1994.

### Patients and methods

### *Patients*

Preoperative serum samples were obtained from 179 consecutive patients with verified pancreatic cancer. Patients were classified according to the UICC TNM classification: 40 patients had stage I disease, 54 patients stage II-III disease and 85 patients stage IV disease. Most of the patients with stage II-III disease underwent a palliative operation. In these operations, local lymph nodes were not always removed adequately to allow differentiation between stages II and III. Therefore, patients with stage II and III tumours, representing locally spread, non-resectable disease, were combined for analysis. Histological type and differentiation grade were determined when histological specimens were available.

For the control group, serum samples were collected from 112 patients with benign diseases that might cause symptoms and signs that resemble those of pancreatic cancer. Benign pancreatic disease was found in 42 patients: 22 patients with acute pancreatitis and 20 with chronic pancreatitis. The patients with benign biliary tract diseases (43 patients) included 32 patients with stones in the common bile duct and 11 patients with gall bladder stones, three of whom had acute cholecystitis. All but four patients with common bile duct stones showed various degrees of cholestasis. Sera from 27 patients with benign liver diseases were tested: 17 patients had acute viral hepatitis, four chronic hepatitis, three hepatic cirrhosis, one acute alcoholic hepatitis, one primary biliary cirrhosis and one benign adenoma.

### *Assays*

The serum samples were frozen and stored at $-20°C$ or $-70°C$ before the assays. Serum CA 242 levels were measured by a dissociation-enhanced lanthanide fluoroimmunoassay (DELFIA) (Wallac Oy, Turku, Finland) as previously described (Nilsson *et al.*, 1988, 1992; Kuusela *et al.*, 1991). An upper limit of normal of 20 U $ml^{-1}$, corresponding to the 99.4 percentile of healthy blood donors, has been recommended for the assay (Nilsson *et al.*, 1988). CA 19-9, CEA (Abbot-Diagnostics, Chicago, IL, USA) and CA 50 (Pharmacia Diagnostics, Uppsala, Sweden) were quantitated by solid-phase radioimmunoassays. Cut-off values of 37 U $ml^{-1}$, 5 ng $ml^{-1}$ and 17 U $ml^{-1}$, respectively, were used. The inter-assay variations of the tests were below 5%, and the intra-assay variations below 6%.

### *Statistical methods*

For the comparison of various tumour markers, cut-off values representing the 90% and 95% specificity levels of patients with relevant benign diseases were determined. The correlation between the CA 242 and CA 19-9 concentrations was calculated by linear regression using the logarithms of the serum levels. Differences in mean values were calculated using the Mann-Whitney *U*-test for non-paired samples. Receiver operating characteristic (ROC) curves were constructed by calculating the true-positive fraction (sensitivities) and false-positive fraction (specificities) of the CA 242 and CA 19-9 assays at several cut-off points. ROC analysis is a graphical method of comparing the sensitivity and specificity of different diagnostic tests (Metz, 1978).

### Results

### *CA 242 in pancreatic cancer*

The serum level of CA 242 was higher than 20 U $ml^{-1}$ in 133 out of 179 patients with pancreatic cancer (74%) (Figure 1, Table I). The median value was 141 U $ml^{-1}$, mean 25,822 U $ml^{-1}$ and range 0-2,166,000 U $ml^{-1}$. Of these patients, 94 (53%) had a level higher than 111 U $ml^{-1}$, which was the highest value found in patients with benign diseases. CA 242 was elevated in 22 out of 40 patients with stage I disease (55%), in 45 out of 54 patients with stage II-III disease (83%) and in 66 out of 85 patients with stage IV disease (78%). The CA 242 level was significantly higher in stage II-III ($P = 0.02$) and in stage IV patients ($P = 0.002$) than in patients with stage I disease. The difference between stage II-III and stage IV patients was not significant ($P = 0.24$). The CA 242 level in patients with pancreatic cancer was significantly higher than in patients with benign diseases ($P = 0.0001$). This was also true when comparing different stages of pancreatic cancer with benign diseases ($P = 0.0001$).

### *CA 242 in benign diseases*

The serum concentration of CA 242 was increased above the cut-off level of 20 U $ml^{-1}$ in 10 out of 112 patients (9%) with benign diseases of the pancreas, biliary tract and liver (Figure 1, Table I). The median value was 6 U $ml^{-1}$, the mean value 10 U $ml^{-1}$ and the highest value 111 U $ml^{-1}$. Five out of 43 patients with benign biliary diseases (15%) had an increased serum concentration (median 6 U $ml^{-1}$, mean 12 U $ml^{-1}$, range 0-111 U $ml^{-1}$). All five patients had common bile duct stones with cholestasis. Of 42 patients with benign pancreatic diseases, elevated CA 242 levels were seen in three patients with chronic pancreatitis, but in none with acute pancreatitis (7%; median 6 U $ml^{-1}$, mean 10 U $ml^{-1}$, range 0-46 U $ml^{-1}$). Of 27 patients with benign liver diseases, two patients with acute viral hepatitis had a slightly elevated CA 242 level (7%; median 4 U $ml^{-1}$, mean 8 U $ml^{-1}$, range 0-40 U $ml^{-1}$).

### *CA 19-9 in pancreatic cancer*

The serum CA 19-9 concentration was higher than 37 U $ml^{-1}$ in 148 out of 179 patients with pancreatic cancer (82.7%) (Figure 1, Table I). The median value was 560 U $ml^{-1}$, mean 22,402 U $ml^{-1}$ and range 0-1,858,900 U $ml^{-1}$. Of these patients 63 (35%) had a level higher than 1,400 U $ml^{-1}$, which was the highest value of the benign group. CA 19-9 was elevated in 27 out of 40 patients with stage I disease (68%), in 49 out of 54 patients with stage II-III disease

**Figure 1** Serum CA 242 concentrations in patients with pancreatic cancer, and with benign pancreatic, biliary and liver diseases. The cut-off value for the CA 242 test is marked with a dashed line.

**Table I** Comparison of CA 242 and CA 19-9 in patients with pancreatic cancer and patients with benign pancreatic, biliary and liver diseases, using the recommended cut-off levels and cut-off levels representing the 90% and 95% specificity levels

| | *No.* | *CA 242* >*20 U ml*$^{-1}$ (%) | *CA 19-9* >*37 U ml*$^{-1}$ (%) | *CA 242* >*19.5 U ml*$^{-1}$ (%) | *CA 19-9* >*60 U ml*$^{-1}$ (%) | *CA 242* >*27 U ml*$^{-1}$ (%) | *CA 19-9* >*111 U ml*$^{-1}$ (%) |
|---|---|---|---|---|---|---|---|
| Pancreatic cancer | 179 | 74 | 83 | 75 | 78 | 70 | 72 |
| Stage I | 40 | 55 | 68 | 58 | 65 | 50 | 60 |
| Stage II-III | 54 | 83 | 91 | 83 | 83 | 78 | 76 |
| Stage IV | 85 | 78 | 85 | 78 | 78 | 74 | 75 |
| Benign diseases | 112 | 9 | 19 | 10 | 10 | 5 | 5 |
| Benign pancreatic diseases | 42 | 7 | 12 | 10 | 5 | 5 | 2 |
| Benign biliary diseases | 43 | 15 | 28 | 14 | 19 | 5 | 12 |
| Benign liver diseases | 27 | 7 | 15 | 7 | 4 | 7 | 0 |

(91%) and in 72 out of 85 patients with stage IV disease (85%). The CA 19-9 level was significantly higher in stage II–III ($P = 0.007$) and in stage IV patients ($P = 0.001$) than in patients with stage I disease. The difference between stage II–III and stage IV patients was non-significant ($P = 0.33$). The CA 19-9 level in all stages of pancreatic cancer was significantly higher than in patients with benign diseases ($P = 0.0001$).

### *CA 19-9 in benign diseases*

The serum concentration of CA 19-9 was increased above the cut-off level of 37 U ml$^{-1}$ in 21 out of 112 patients with benign diseases of the pancreas, biliary tract and liver (19%; median 12 U ml$^{-1}$, mean 41 U ml$^{-1}$, range 0–1,400 U ml$^{-1}$) (Figure 1, Table I). The CA 19-9 level was elevated in 12 out of 43 patients with benign biliary diseases (28%; median 16 U ml$^{-1}$, mean 77 U ml$^{-1}$, range 0–1,400 U ml$^{-1}$). Nine of these patients had common bile duct stones with various degree of extrahepatic cholestasis, one patient had acute cholecystitis and two patients had gall bladder stones without cholecystitis. In benign pancreatic diseases CA 19-9 was elevated in three patients with chronic and two patients with acute pancreatitis (12%; median 10 U ml$^{-1}$, mean 20 U ml$^{-1}$, range 0–185 U ml$^{-1}$). Four patients with benign liver disease (15%) had an elevated CA 19-9 serum level: two with viral hepatitis, one with cirrhosis, and one with acute alcoholic hepatitis.

### *Comparison of CA 242 and CA 19-9*

There was a high correlation between the CA 242 and CA 19-9 levels in serum ($r^2 = 0.732$). The sensitivity of the CA 19-9 test for pancreatic cancer was higher than that of the CA 242 test in all stage groups, but the specificity was clearly lower. When comparing the two markers by ROC curve analysis, the curves were nearly identical (Figure 2). At the 90% specificity level, the sensitivity of CA 242 was 75% and that of CA 19-9 78%. The corresponding cut-off levels were 19.5 U ml$^{-1}$ and 60 U ml$^{-1}$ respectively. At the 95% specificity level, the sensitivities for CA 242 and CA 19-9 were 70% and 72%, respectively, and the cut-off values were 27 U ml$^{-1}$ and 111 U ml$^{-1}$ respectively (Table I).

Using the recommended cut-off values, two patients with pancreatic cancer had an elevated CA 242 level but a normal CA 19-9 level, while the opposite was true in 17 patients. If elevation of either CA 242 or CA 19-9 was considered a positive result, the overall sensitivity of the combination of

**Figure 2** ROC curves for CA 242 (——) and CA 19-9 (---) in pancreatic cancer. The true-positive fraction was calculated from 179 patients with pancreatic cancer, and the false-positive fraction from 112 patients with benign pancreatic, biliary and liver diseases.

the markers was 84%. If both markers were required to be elevated for a positive test result, the overall sensitivity was 73%. The corresponding specificities were 80% and 93% respectively.

Eight out of ten patients with benign diseases with elevated CA 242 level also had an elevated CA 19-9 level. The highest serum levels of CA 19-9 were 1,400, 410 and 365 U ml$^{-1}$, and the corresponding CA 242 levels 58, 3 and 26 U ml$^{-1}$ respectively. The highest CA 242 levels were 111, 58 and 46 U ml$^{-1}$ and the corresponding CA 19-9 levels 126, 1,400 and 46 U ml$^{-1}$. The assay parameters for the CA 242 and CA 19-9 assays, and for the combinations of the tests, are summarised in Table II.

### *Comparison of serum CA 242, CA 19-9 and histological grade*

The serum level of CA 242 was correlated with the histological differentiation grade in 111 patients: 78 with well-or moderately differentiated adenocarcinoma and 33 with poorly differentiated or anaplastic carcinoma. When analysing all patients no difference was seen between the groups. When patients were divided according to stage, there was a significant difference in stage IV. Patients with well- or moderately differentiated tumours had a higher serum CA 242 level than patients with poorly differentiated tumours ($P = 0.04$). A difference was also seen for CA 19-9, but it was not significant. In stage I and stage II–III, patients with poorly differentiated carcinomas had slightly higher serum CA 242 and CA 19-9 levels than patients with well- or moderately differentiated carcinomas, but the number of patients with poorly differentiated tumours was very small and the difference was not significant.

### *Comparison of CA 242 with CA 50 and CEA*

The CA 50 level was available in 72 patients with pancreatic cancer and in 67 patients with benign diseases. The CEA concentration was available in 156 and 69 patients respectively. Using linear regression there was a high correlation between CA 242 and CA 50 ($r^2 = 0.846$), but no correlation between CA 242 and CEA ($r^2 = 0.314$). When comparing CA 242 with CA 50 and CEA by ROC curve analysis, CA 242 had a higher sensitivity than CA 50 at every specificity level, and a higher sensitivity than CEA at all specificity levels higher than 70% (Figures 3 and 4).

If elevation of both CA 242 (>20 U ml$^{-1}$) and CEA (>5 ng ml$^{-1}$) was considered a positive test result, only 33% showed a positive test. If elevation of either CA 242 or CEA was considered a positive result, the sensitivity of the combination of the markers was 85%. The corresponding specificity was 81%. At this specificity level the sensitivity of the CA 242 test alone was 78%.

### *Comparison of CA 242 and CA 19-9 according to serum bilirubin level*

Patients with benign disease and an elevated serum bilirubin level (>20 μmol l$^{-1}$) had significantly higher mean serum CA 19-9 concentration than patients with a normal bilirubin level ($P < 0.05$). No difference was seen for CA 242 ($P = 0.99$). In

**Table II** Assay parameters for the CA 242 and the CA 19-9 assays in pancreatic cancer

| *Assay parameter* | *CA 242 (%)* | *CA 19-9 (%)* |
|---|---|---|
| Sensitivity | 74 | 83 |
| Specificity | 91 | 81 |
| Positive predictive value | 93 | 88 |
| Negative predictive value | 70 | 75 |

Cut-off values: CA 242, 20 U ml$^{-1}$; CA 19-9, 37 U ml$^{-1}$. Sensitivity = TP/(TP + FN); specificity = TN/(TN + FP); positive predictive value = TP/(TP + FP); negative predictive value = TN/(TN + FN);. TP, true positive; FN, false negative; TN, true negative; FP, false positive.

**Figure 3** ROC curves for CA 242 (——) and CA 50 (---) in pancreatic cancer. The true-positive fraction was calculated from 72 patients with pancreatic cancer, and the false-positive fraction from 67 patients with benign pancreatic, biliary and liver diseases.

**Figure 4** ROC curves for CA 242 (——) and CEA (---) in pancreatic cancer. The true-positive fraction was calculated from 156 patients with pancreatic cancer, and the false-positive fraction from 69 patients with benign pancreatic, biliary and liver diseases.

cancer patients, the difference between patients with elevated versus normal bilirubin level was not significant in any stage group. CA 19-9: stage I, $P = 0.78$; stage II–III, $P = 0.13$; stage IV, $P = 0.10$. CA 242: stage I, $P = 0.47$; stage II–III, $P = 0.45$; stage IV, $P = 0.39$.

Using linear regression there was no correlation between the serum levels of tumour markers CA 242/CA 19-9 and serum bilirubin ($r^2 = 0.041/0.11$) and alkaline phosphatase ($r^2 = 0.045/0.07$).

### Discussion

During the 10 years that CA 19-9 has been available for clinical use (Del Villano *et al.*, 1983), it has gained a position as reference marker for pancreatic cancer, with which new markers are compared. Only some years after the presentation of CA 19-9, another tumour marker CA 50, recognising the same antigenic determinant, the sialylated Lewis$^a$ antigen, was described (Lindholm *et al.*, 1983). In serum, both markers are associated with a high molecular weight carbohydrate-rich mucin fraction (Lindholm *et al.*, 1983; Magnani *et al.*, 1983). Johansson *et al.* (1991*a*, *b*) have demonstrated coexpression of CA 50 and sialylated Lewis$^a$, i.e. CA 19-9, on the same macromolecular complex, which they call CanAg. This macromolecule also expresses other antigens, including CA 242, a carbohydrate different from CA 19-9 and CA 50 (Nilsson *et al.*, 1992).

CA 242 is defined by monoclonal antibody C 242, obtained by the same immunisation procedure as the C 50 antibody (Lindholm *et al.*, 1985). Different combinations of antibodies were studied in order to achieve optimal sensitivity and specificity for the macromolecule expressing these antigens (Johansson *et al.*, 1991*b*). An assay using an antibody against sialylated Lewis$^a$ as catcher and the C 242 antibody as detector antibody showed better specificity for cancer than the CA 50 assay, which uses C 50 as both catcher and detector antibody. This assay has been introduced as the CA 242 marker test.

Preliminary results on the serum expression of CA 242 in pancreatic cancer were described in 1991 by Kuusela *et al.* Later other small series including 24–68 patients were reported (Nilsson *et al.*, 1992; Pasanen *et al.*, 1992; Banfi *et al.*, 1993; Röthlin *et al.*, 1993). The results were promising and CA 242 has shown sensitivities and specificities similar to those of CA 19-9 and CA 50. For this study we collected a large series of 179 patients with pancreatic cancer, enabling division of patients in stage groups according to the UICC TNM classification.

Our study confirms previous observations that the assay parameters of CA 242 are similar to those of CA 19-9 in the primary diagnosis of pancreatic cancer (Kuusela *et al.*, 1991; Banfi *et al.*, 1993; Röthlin *et al.*, 1993). The highest serum levels of CA 242 and CA 19-9 were found in patients with unresectable disease, which can be diagnosed by other clinical, radiological and laboratory investigations rather easily. In stage I disease, in which surgical treatment with curative intent is possible, more than half of the patients had elevated CA 242 and CA 19-9 levels at the 90% specificity level (58% and 65% respectively). If five patients with resectable stage II–III tumours were included, 60% of patients with resectable disease had an elevated CA 242 level, and 67% an elevated CA 19-9 level.

There was a high correlation between the serum levels of CA 242 and CA 19-9. Using ROC curve analysis the results were nearly identical. Usually both markers were elevated in the same patients, and in most patients with either marker elevated and the other normal the difference in serum concentration was small. However, a small number of patients had a high CA 242 serum concentration but only slightly elevated CA 19-9 or vice versa. These patients show that, in spite of the similarity of the markers, there are in some patients clear differences in the expression of the antigens. The reason for this is still not known.

When comparing the serum CA 242 level with the histological differentiation grade, stage IV patients with well- to moderately differentiated carcinomas had significantly higher CA 242 levels than patients with poorly differentiated carcinomas. This is in concordance with immunohistochemical findings showing clearly stronger expression of CA 242 in well- to moderately differentiated tumours than in poorly differentiated or anaplastic carcinomas (Haglund *et al.*, 1989). In stage I and II–III patients, poorly differentiated tumours seemed to be associated with slightly higher CA 242 levels than well- to moderately differentiated tumours. However, the number of patients with poorly differentiated tumours in these stage groups was too small for definite conclusions to be drawn.

Because of the high correlation between the test results there was no advantage in combining CA 242 with CA 19-9 or CA 50. No correlation was seen between CA 242 and CEA. A combination of CA 242 and CEA increased the sensitivity by 7% at the 81% specificity level. Combination of CA 19-9 and CEA increased the sensitivity by 4% compared with CA 19-9 alone. However, the clinical utility of combining the tests is limited by the fact that the majority of patients with elevated CEA and normal CA 242 or CA 19-9 had disseminated disease.

The highest frequency of false-positive CA 19-9 levels has been described in patients with extrahepatic jaundice (Jalanko *et al.*, 1984; Haglund *et al.*, 1986; Steinberg *et al.*, 1986), and this was confirmed in our study. In this patient group there was a difference between CA 19-9 and CA 242 in favour of CA 242. Although elevated CA 242 levels were found in some patients with common bile duct stones, the frequency of elevated levels was lower, and the increase in the serum concentration smaller than that of CA 19-9. When analysing all patients with benign diseases, significantly higher CA 19-9 levels were seen in jaundiced compared with non-jaundiced patients, whereas no significant difference was seen for CA 242. The highest CA 19-9 serum concentrations have been reported in patients with cholangitis (Albert *et al.*, 1988). In the present study, one patient with a CA 19-9 value of 1,400 U $ml^{-1}$ had cholangitis. The corresponding CA 242 serum level was only 58 U $ml^{-1}$. A larger series of patients with cholangitis should be collected to further evaluate the potential advantage of CA 242 over CA 19-9.

At the time of diagnosis, most patients with pancreatic cancer show extrahepatic cholestasis with elevated serum levels of bilirubin and alkaline phosphatase. It seems obvious that cholestasis contributes to elevated marker levels in these patients. In stage II–IV cancer patients, the mean values of CA 19-9 and CA 242 appeared higher in jaundiced than in non-jaundiced patients, although the differences were not significant. The *P*-values were lower for CA 19-9 than for CA 242, supporting the findings seen in benign diseases. In clinical practice, biliary decompression has been found to reduce the CA 242 and CA 19-9 levels to a variable degree (C. Haglund *et al.*, unpublished data). In spite of the higher frequency of elevated marker levels in cancer patients with cholestasis, there was no clear-cut correlation between the serum levels of these two tumour markers in malignant or benign diseases and the serum levels of bilirubin or alkaline phosphatase. This may be explained by the fact that a minority of tumours do not express CA 242 or CA 19-9 (Haglund *et al.*, 1989), and these patients have a normal serum level even though they might be jaundiced. On the other hand, some patients have high tumour production and strong serum expression of these antigens without obstruction of the common bile duct.

Preoperatively, chronic pancreatitis can sometimes be very difficult to differentiate from pancreatic cancer. In these patients tumour markers might be helpful. Only three out of 20 patients with chronic pancreatitis had an elevated CA 242 level, but none of the patients with acute pancreatitis did. The highest value found in patients with chronic pancreatitis was 46 U $ml^{-1}$. Hence, in this series a clearly elevated tumour marker level strongly indicated malignant disease. On the other hand, half of the patients with stage I tumours and 31% of patients with stage II–III tumours had a CA 242 serum level below 46 U $ml^{-1}$. Similarly, 55% and 28% of stage I and stage II–III patients, respectively, had a CA 19-9 level below 185 U $ml^{-1}$, which was the highest value found in patients with pancreatitis.

Benign liver disease is frequently associated with elevated CA 19-9, CA 50 and CEA levels (Jalanko *et al.*, 1984; Chan *et al.*, 1985; Steinberg *et al.*, 1986). In this study 4 of 27 patients had a slightly elevated CA 19-9 level, but only two patients with acute viral hepatitis had an elevated CA 242 level. Our material included only a few patients with chronic liver disease, and further studies are needed to evaluate the possible influence of chronic liver failure on the CA 242 level.

In conclusion, CA 19-9 has in many studies and in clinical practice been shown to be a useful complement to other diagnostic methods in symptomatic patients with pancreatic cancer. An elevated tumour marker level may lead to an intensified search for pancreatic cancer. In this study we report the results on a novel tumour marker, CA 242, which are similar to those of CA 19-9 in the primary diagnosis of pancreatic cancer. In clinical work, the higher specificity when using the recommended cut-off levels of the tests is a clear advantage of CA 242 compared with CA 19-9. Studies comparing CA 242 and CA 19-9 in follow-up and as prognostic markers are ongoing, and will help to decide whether CA 242 might replace CA 19-9 as the standard marker for pancreatic cancer. CA 242 also seems to be very promising in the diagnosis of colorectal cancer (Kuusela *et al.*, 1991; Nilsson *et al.*, 1992). If CA 242 becomes a routine marker for colorectal cancer, it would seem to be convenient to use the same marker in patients with pancreatic cancer.

The authors thank Wallac Oy for kindly supplying the CA 242 assay. This study has been supported by grants from Medicinska understödsföreningen Liv och Hälsa, the Karin and Einar Stroems Foundation and Finska Läkaresällskapet.

### References

ALBERT, M.B., STEINBERG, W.M & HENRY, J.P. (1988). Elevated serum levels of tumor marker CA 19-9 in acute cholangitis. *Digest. Dis. Sci.*, **33**, 1223–1225.

BANFI, G., ZERBI, A., PASTORI, S., PAROLINI, D., DI CARLO, V. & BONINI, P. (1993). Behavior of tumor markers CA 19.9, CA 195, CAM 43, CA 242, and TPS in the diagnosis and follow-up of pancreatic cancer. *Clin. Chem.*, **39**, 420–423.

BENINI, L., CAVALLINI, G., ZORDAN, D., RIZZOTTI, P., RIGO, L., BROCCO, G., PEROBELLI, L., ZANCHETTA, M., PEDERZOLI, P. & SCURO, L.A. (1988). A clinical evaluation of monoclonal (CA 19-9, CA 50, CA 125) and polyclonal (CEA, TPA) antibody-defined antigens for the diagnosis of pancreatic cancer. *Pancreas*, **3**, 61–66.

CHAN, S.H., LINDHOLM, L., WONG, L. & OON, C.J. (1985). Tumor markers in hepatocellular carcinoma in Singaporean Chinese. In *Tumor Markers Antigens*, Holmgren, J. (ed.) pp. 106–113. Studentlitteratur: Lund, Sweden.

DEL VILLANO, B.C., BRENNAN, S., BROCK, P., BUCHER, C., LIU, V., MCCLURE, M., RAKE, B., WESTRICK, B., SCHOEMAKER, H. & ZURAWSKI, V.R. (1983). Radioimmunometric assay for a monoclonal antibody-defined tumor marker, CA-19-9. *Clin. Chem.*, **29**, 549–552.

HAGLUND, C., ROBERTS, P.J., KUUSELA, P., SCHEININ, T.M., MÄKELÄ, O. & JALANKO, H. (1986). Evaluation of CA 19-9 as a serum tumour marker in pancreatic cancer. *Br. J. Cancer*, **53**, 197–202.

HAGLUND, C., KUUSELA, P., JALANKO, H. & ROBERTS, P.J. (1987). Serum CA 50 as a tumor marker in pancreatic cancer: a comparison with CA 19-9. *Int. J. Cancer*, **39**, 477–481.

HAGLUND, C., LINDGREN, J., ROBERTS, P.J., KUUSELA, P. & NORDLING, S. (1989). Tissue expression of the tumour-associated antigen CA 242 in benign and malignant pancreatic lesions. A comparison with CA 50 and CA 19-9. *Br. J. Cancer*, **60**, 845–851.

HARMENBERG, U., WAHREN, B. & WIECHSEL, K.-L. (1988). Tumor markers carbohydrate antigens CA 19-9 and CA 50 and carcinoembryonic antigen in pancreatic cancer and benign diseases of the pancreaticobiliary tract. *Cancer Res.*, **48**, 1985–1988.

JALANKO, H., KUUSELA, P., ROBERTS, P., SIPPONEN, P., HAGLUND, C. & MÄKELÄ, O. (1984). Comparison of a new tumour marker, CA 19-9™, with alpha-fetoprotein and carcinoembryonic antigen in patients with upper gastrointestinal diseases. *J. Clin. Pathol.*, **37**, 218–222.

JOHANSSON, C., NILSSON, O., BAECKSTRÖM, D., JANSSON, E.-L. & LINDHOLM, L. (1991*a*). Novel epitopes on the CA50-carrying antigen: chemical and immunochemical studies. *Tumor Biol.*, **12**, 159–170.

JOHANSSON, C., NILSSON, O. & LINDHOLM, L. (1991*b*). Comparison of serological expression of different epitopes on the CA50-carrying antigen CanAg. *Int. J. Cancer*, **48**, 757–763.

KOPROWSKI, H., STEPLEWSKI, Z., MITCHELL, K., HERLYN, M., HERLYN, D. & FULNER, P. (1979). Colorectal carcinoma antigens detected by hybridoma antibodies. *Somat. Cell Genet.*, **5**, 957-972.

KUUSELA, P., HAGLUND, C. & ROBERTS, P.J. (1991). Comparison of a new tumour marker CA 242 with CA 19-9, CA 50 and carcinoembryonic antigen (CEA) in digestive tract diseases. *Br. J. Cancer*, **63**, 636-640.

LINDHOLM, L., HOLMGREN, J., SVENNERHOLM, L., FREDMAN, P., NILSSON, O., PERSSON, B., MYRVOLD, H. & LAGERGÅRD, T. (1983). Monoclonal antibodies against gastrointestinal tumour-associated antigens isolated as monosialogangliosides. *Inst. Arch. Allergy Appl. Immun.*, **71**, 178-181.

LINDHOLM, L., JOHANSSON, C., JANSSON, E.-L., HALLBERG, C. & NILSSON, O. (1985). An immunometric assay (IRMA) for the CA 50 antigen. In *Tumor Marker Antigens*, Holmgren, J. (ed.) pp. 122-133, Studentlitteratur: Lund, Sweden.

MAGNANI, J.L., NILSSON, B., BROCKHAUS, M. ZOPF, D., STEPLEWSKI, Z., KOPROWSKI, H. & GINSBURG, V. (1982). A monoclonal antibody-defined antigen associated with gastrointestinal cancer is a ganglioside containing sialylated lacto-N-fucopentaose II. *J. Biol. Chem.*, **257**, 14365-14369.

MAGNANI, J.L., STEPLEWSKI, Z., KOPROWSKI, H. & GINSBURG, V. (1983). Identificaton of the gastrointestinal and pancreatic cancer-associated antigen detected by monoclonal antibody 19-9 in the sera of patients as a mucin. *Cancer Res.*, **43**, 5489-5492.

METZ, C.E. (1978). Basic principles of ROC analysis. *Semin. Nucl. Med.*, **8**, 283-298.

MÅNSSON, J.E., FREDMAN, P., NILSSON, O., LINDHOLM, L., HOLMGREN, J. & SVENNERHOLM, L. (1985). Chemical structure of carcinoma ganglioside antigens defined by monoclonal antibody C-50 and some allied gangliosides of human pancreatic adenocarcincoma. *Biochim. Biophys. Acta*, **834**, 110-117.

NILSSON, O., MÅNSSON, J.-E., LINDHOLM, L., HOLMGREN, J. & SVENNERHOLM, L. (1985). Sialosyllactotetraosylceramide, a novel ganglioside antigen detected in human carcinomas by a monoclonal antibody. *FEBS Lett.*, **182**, 398-402.

NILSSON, O., JANSSON, E.-L., JOHANSSON, C. & LINDHOLM, L. (1988). CA 242, a novel tumour-associated carbohydrate antigen with increased tumour specificity and sensitivity. *J. Tumor Marker Oncol.*, **3**, 314.

NILSSON, O., JOHANSSON, C., GLIMELIUS, B., PERSSON, B., NØRGAARD-PEDERSEN, B., ANDRÉN-SANDBERG, Å, & LINDHOLM, L. (1992). Sensitivity and specificity of CA 242 in gastrointestinal cancer. A comparison with CEA, CA 50 and CA 19-9. *Br. J. Cancer*, **65**, 215-221.

PASANEN, P.A., ESKELINEN, M., PARTANEN, K., PIKKARAINEN, P., PENTTILÄ, I. & ALHAVA, E. (1992). Clinical evaluation of a new serum tumour marker CA 242 in pancreatic cancer, *Br. J. Cancer*, **65**, 731-734.

RÖTHLIN, M.A., JOLLER, H. & LARGIADER, F. (1993). CA 242 is a new tumor marker for pancreatic cancer. *Cancer*, **71**, 701-707.

STEINBERG, W.M., GELFAND, R., ANDERSON, K.K., GLENN, J., KURZMAN, S.H., SINDELAR, W.F. & TOSKES, P.P. (1986). Comparison of the sensitivity and specificity of the CA 19-9 and carcinoembryonic antigen assays in detecting cancer of the pancreas. *Gastroenterology*, **90**, 343-379.